# A novel mutation in homeobox DNA binding domain of *HOXC13* gene underlies pure hair and nail ectodermal dysplasia (ECTD9) in a Pakistani family

**DOI:** 10.1186/s12881-017-0402-y

**Published:** 2017-04-12

**Authors:** Anwar Kamal Khan, Noor Muhammad, Abdul Aziz, Sher Alam Khan, Khadim Shah, Abdul Nasir, Muzammil Ahmad Khan, Saadullah Khan

**Affiliations:** 1 Department of Biotechnology and Genetic Engineering, Kohat University of Science and Technology (KUST), Kohat, 26000 Khyber Pakhtunkhwa Pakistan; 2 Department of Bioinformatics, Khushal Khan Khattak University, Karak, Pakistan; 3 Department of Biochemistry, Faculty of Biological Sciences, Quaid-i-Azam University, Islamabad, 45320 Pakistan; 4 Gomal Centre of Biochemistry & Biotechnology, Gomal University, D.I.Khan, Pakistan

**Keywords:** PHNED, *HOXC13*, Mutation, Pakistani family

## Abstract

**Background:**

Pure hair and nail ectodermal dysplasia (PHNED) is a congenital disorder of hair abnormalities and nail dysplasia. Both autosomal recessive and dominant inheritance fashion of PHNED occurs. In literature, to date, five different forms of PHNED have been reported at molecular level, having three genes known and two loci with no gene yet.

**Methods:**

In this study, a four generations consanguineous family of Pakistani origin with autosomal recessive PHNED was investigated. Affected members exhibited PHNED phenotypes with involvement of complete hair loss and nail dysplasia. To screen for mutation in the genes (*HOXC13*, *KRT74*, *KRT85*), its coding exons and exons-intron boundaries were sequenced. The 3D models of normal and mutated HOXC13 were predicted by using homology modeling.

**Results:**

Through investigating the family to known loci, the family was mapped to ectodermal dysplasia 9 (ECTD9) loci with genetic address of 12q13.13. Mutation screening revealed a novel missense mutation (c.929A > C; p.Asn310Thr) in homeobox DNA binding domain of *HOXC13* gene in affected members of the family. Due to mutation, loss of hydrogen bonding and difference in potential energy occurs, which may resulting in alteration of protein function.

**Conclusion:**

This is the first mutation reported in homeodomain, while 5^th^ mutation reported in *HOXC13* gene causing PHNED.

## Background

Pure hair and nail ectodermal dysplasia (PHNED; MIM 602032) is a rare genetic condition, characterized by sparse to complete absence of hairs and nail dystrophy without involvement of any other ectodermal and non-ectodermal manifestations. Hair abnormalities range its mild hair loss to complete absence on the scalp, or may be sparse/absence on rest of the body parts like eyebrows, eyelashes, axillary hair and pubic hair. Nail dystrophy affecting mostly all 20 nails varying in phenotypes including irregular, fragile and spoon shaped.

Inheritance mode of PHNED is both autosomal recessive and dominant [1, 2]. No gene has been reported for autosomal dominant PHNED. Based on OMIM classification, there are five autosomal recessive pure hair and nail ectodermal dysplasia types have been reported. These includes ectodermal dysplasia hair and nail type 4 (ECTD4; MIM 602032) lying on chromosome 12q13.13 with mutations in *KRT85* gene [3], ectodermal dysplasia hair and nail type 5 (ECTD5; MIM 614927) located on chromosome 10q24.32-q25.1 [4], ectodermal dysplasia hair and nail type 6 (ECTD6; MIM 614928) with chromosomal address 17p12-q21.2 [5], ectodermal dysplasia hair and nail type 7 (ECTD7; MM 614929) lying on chromosome 12q13.13 with mutation in *KRT74* gene [6] and ectodermal dysplasia hair and nail type 9 (ECTD9; MIM 614931) harboring *HOXC13* gene on chromosome 12q13.13 [7–9].

*KRT85* and *KRT74* genes mutations have been reported only in Pakistani families, while *HOXC13* gene mutations have been reported in few other ethnicities including Chinese, Afghani and Syrian along with Pakistani families. Both ECTD5 and ECTD6 linked families are also of Pakistani origin, however the causative genes are yet to be known.

In the present study, a consanguineous Pashto speaking origin family was recruited from Bannu district of Khyber Pakhtunkhwa province of Pakistan, segregating autosomal recessive form of PHNED. Genotyping data and sequence analysis identified a novel missense mutation (c.929A > C) in *HOXC13* gene.

## Methods

**Fig. 1:**
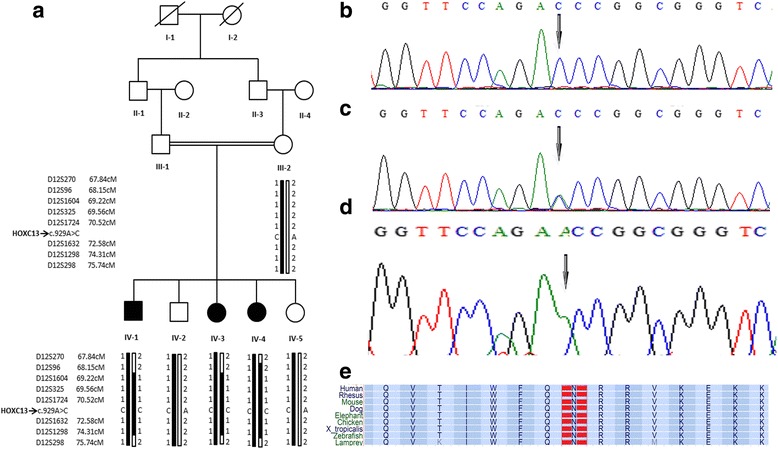
Pedigree and haplotype of the family (**a**). Sequence analysis of the *HOXC13* gene showing homozygous affected member (**b**), heterozygous carrier (**c**) and homozygous unaffected member (**d**). Clustal alignment of HOXC13 shows complete evolutionary conservation of the Asn310 residue (*shaded*) in all species with known ortholog (**e**)

In the present investigation, a consanguineous Pakistani family of Pashtun origin with PHNED was recruited (Fig. 1a). To undertake the study, ethical approval was obtained from the Institutional Review Board (IRB) of Kohat University of Science & Technology (KUST), Khyber Pakhtunkhwa, Pakistan. The study was conducted after obtaining written informed consent from all participating subjects including affected individuals, parents and other normal members of the family.

Blood samples were collected from six individuals including three affected members including a male (IV-1, age: 37 years) and two females (IV-3, age: 31 years; IV-4, age: 25 years).

DNA was extracted from the blood samples collected from both affected and unaffected members of the family through phenol-chloroform method. For genotyping, highly polymorphic microsatellite markers were used mapped to the flanking region of the *keratin* cluster and *HOXC* cluster genes. After establishment of linkage to the region, based on phenotypes, three genes (*HOXC13*, *KRT74* and *KRT85*) were prefer to sequence in both affected and unaffected individuals. Big Dye Terminator Cycle Sequencing Kit v3.1 (Applied Biosystems, CA USA) was used for sequencing and the product was analyzed on genetic analyzer ABI 3500 (Applied Biosystems, Inc., Foster City, CA, USA). Primer3 software [http://frodo.wi.mit.edu/primer3/] was used to design primer sequences. Sequence variants in the affected individuals were identified by BIOEDIT sequence alignment (editor version 6.0.7; lbis Biosciences Inc., Carlsbad, CA, USA). PolyPhen-2 [http://genetics.bwh.harvard.edu/pph/] was used to predict the possible effect of the mutation on function of protein. The protein sequences of the pathogenic sequence variant and the evolutionary conservation of amino acid was deduced through multiple sequence alignment tool ClustalW [www.ebi.ac.uk/clustalw/].

### Protein modelling

The crystal structure Human HOXC13 protein was not available in PDB. So, Homology modeling techniques were utilized to construct the three dimensional structure of Homeobox domain (241aa-322aa) of human HOXC13. The amino acid sequence of Homeobox domain was retrieved from the NCBI database [http://www.ncbi.nlm.nih.gov/], imported to pBlast search against PDB (Protein data bank). We first constructed the homology model of the wild type using the PDB structure 2L7Z as a template, and then built the mutant structure by mutating the selected residue [10]. The model was built using the Molecular Operating Environment (MOE). A series of 10 independent models for protein was built using the Boltzmann weighted randomized procedure combined with specialized logic for the handling of sequence insertions and deletions [11]. Out of 20 models, the model with best MOE packing score was selected for further mutation analysis. The protein structure was visually inspected using PYMOL viewer [http://www.pymol.org].

## Results

**Fig. 2:**
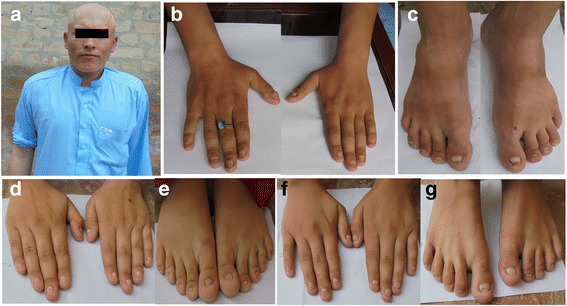
Phenotypes of affected members. Complete absence of hairs on scalp, eyebrows, eyelashes and rest of the body of individual IV-1 (**a**). Note dystrophic, irregularly shaped nails at the distal portion and distal onycholysis of the digits on hands and toe nails dystrophy in affected members IV-1 (**b**, **c**), IV-3 (**d**, **e**) and IV-4 (**f**, **g**)

In the present investigation, three individuals in the family were affected including a brother and two sisters. They were born to healthy first cousin parents. All affected members of the family showed complete hair loss on scalp and rest of the body (Fig. 2a). Affected brother and sisters have dystrophic, irregularly shaped nails at the distal portion and distal onycholysis of the digits on hands while toe nails are dystrophic in all affected members (Fig. 2b-g). Symptoms were present since birth. Other ectodermal structures (teeth and sweat glands) were normal. No other associated abnormalities like mental retardation and skeletal involvement were observed. The parents and other normal siblings show normal hair texture of scalp and rest of the body as well as normal nails.

Based on phenotypes, linkage to the known locus was tested by genotyping highly polymorphic microsatellite markers in six members of the family. All the three affected members showed homozygosity with several markers (D12S1604, D12S325, D12S1724, D12S1632, D12S1298) linked to *Keratin* and *HOXC* genes cluster located on chromosome 12p11.1–q21.1. Three different genes (*KRT74*, *KRT85*, *HOXC13*) have been reported in the linked region to be responsible for PHNED [3, 6, 9].

In order to identify the causative gene responsible for this family harboring PHNED, we sequenced all the reported three genes. Sequence analysis revealed a novel homozygous A to C transversion mutation in *HOXC13* gene at nucleotide position 929 (c.929A > C). This sequence change results in substitution of Aspargine residue with threonine at amino position 310 (p.Asn310Thr) of HOXC13 protein. Pathogenic sequence variant identified here is present in heterozygous conditions in both healthy siblings and a mother. This mutation is a pathogenic, and not present outside the family because we screened 102 ethnically match control individuals. The variant is absent both in “Exome variant server and Exome Aggregation consortium”. No pathogenic sequence variant was identified in *KRT85* and *KRT74*.

**Fig. 3:**
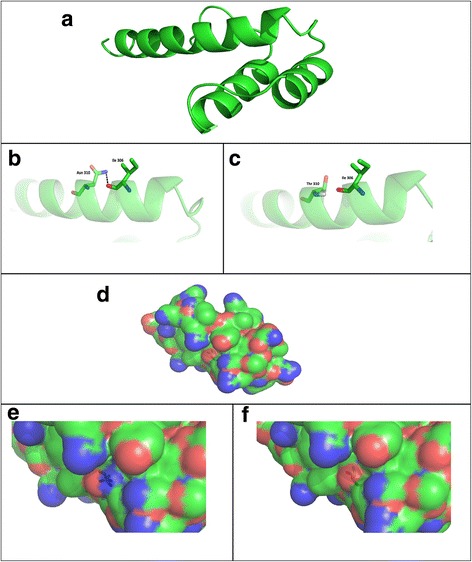
Bioinformatics analysis of wild and mutant HOXC13 protein. **a** Representation of predicted structure for homeobox domain of HOXC13 by means of Molecular Operating Environment (MOE v2013) software package. **b** Representation of wild type and **c** mutant type protein interactions. **d** Computed surface of the homeobox domain of the HOXC13, colored by dystrophicity. **e** Computated surface of wild type and **f** mutant type homeobox domain of HOXC13

The amide Asn residue in the mutant is replaced by Thr residue. The Asn310 in wild type is involved in the hydrogen bonding with nearby Ile306 while the Thr310 loss the contact (Fig. 3a-c). Hence, the mutant is less stable which is supported by increase in potential energy (−1504.414 kcal/mol) as compared to wild type HOXC13 (−1505.945 kcal/mol). As a consequence of this loss of hydrogen bonding, difference in potential energy and nature of amino acids and different surface area may alter the function of the protein (Fig. 3d-f).

## Discussion

The present study describes PHNED in a consanguineous Pakistani family. Clinical features of affected individuals observed in the present family is mostly similar to those reported earlier in ECTD9 families [7–9]. All affected members have complete hair loss and nail dysplasias. To date, five mutations (Tyr130*, Leu119Trpfs∗20, His68Glnfs*84, Ser135*, 27.6 kb deletion) have been reported in this gene causing PHNED [7–9], of these two mutations have been reported in Pakistani families [http://pakmutation.com/nonsyndromicsearch.aspx]. Based on genotyping and subsequently Sanger sequencing data, we identified a novel mutation (c.929A > C; p.Asn310Thr) in *HOXC13* gene.

*HOXC13* gene is composed of two exons spanning 2.423 kbs of genomic DNA with chromosomal address of 12q13.3, while encoding a protein of 330 amino acids (GenBank accession number, NM 017410). It contains a cluster of 61 amino acids (amino acids 258–318) forming the DNA binding homeodomain [http://www.uniprot.org/uniprot/Q86TI1]. This homeodomain is involved in the regulation of transcriptional activity of several hair keratin genes and FOXN1 in hair follicles and nails. The mutated aspargine residue at position 310 (identified in the present family), lying in the homeodomain region is highly conserved in other HOXC13 orthologs (Fig. 1e). Analysis of the protein sequence by protein prediction tool PolyPhen2 [http://genetics.bwh.harvard.edu/pph2/] revealed that the substitution of aspargine by threonine (p.Asn310Thr) could potentially have a damaging effect on HOXC13 structure. The mutation was also tested on mutation taster, predicting disease causing.

*HOXC* gene cluster is located near keratin type II gene cluster. These genes encode highly conserved homeobox family members, which are involved in morphogenesis of several organs. HOXC13 is involved in the development of hair and nails [12]. It also regulates the expression of several other genes (*FOXN1*, *DSG4*, *CRISP1*, *FOXQ1*) involved in hair follicles and/or nail units.

## Conclusion

We have reported a novel mutation in the *HOXC13* gene, results in pure hair and nail dysplasia. A homozygous missense mutation reported here lying in the most conserve homeobox DNA binding domain, which confirms the significant role assigned to HOXC13 in ectodermal development. This study further supports the previously reported findings that homozygous mutations in the *HOXC13* gene cause ECTD9.

## Abbreviations

ECTD9: Ectodermal dysplasia type 9
PHNED: Pure hair nail ectodermal dysplasia
